# Media Bias and Factors Affecting the Impartiality of News Agencies during COVID-19

**DOI:** 10.3390/bs12090313

**Published:** 2022-08-29

**Authors:** Minghua Xu, Ziling Luo, Han Xu, Bang Wang

**Affiliations:** 1 Journalism and Information Communication School, Huazhong University of Science and Technology, Wuhan 430074, China; 2 School of Electronic Information and Communications, Huahzhong University of Science and Technology, Wuhan 430074, China

**Keywords:** COVID-19, sentiment analysis, infodemic, news agencies, media impartiality, BERT

## Abstract

When COVID-19 was raging around the world, people were more fearful and anxious. In this context, the media should uphold impartiality and shoulder the responsibility of eliminating misinformation. Therefore, our research adopted sentiment analysis technologies to analyze the impartiality of news agencies and analyzed the factors that affect the impartiality of COVID-19-related articles about various countries. The SentiWordNet3.0 and bidirectional encoder representations from transformers (BERT) models were employed to analyze the articles and visualize the data. The following conclusions were redrawn in our research. During the pandemic, articles of some news agencies were not objective; the impartiality of news agencies was related to the reliability of news agencies instead of the bias of news agencies; there were obvious differences in the coverage and positivity of international news agencies to report the performance of COVID-19 prevention and control in different countries.

## 1. Introduction

A pandemic caused by the new coronavirus was found in China at the end of 2019 and then swept the world at the beginning of the following year. On 11 February, the Director-General of the World Health Organization (WHO) Tedros Adhanom Ghebreyesus announced in Geneva, Switzerland that pneumonia caused by the new coronavirus was named as “COVID-19” [1,2]. Humanity is collectively facing its most daunting challenge since the Second World War. COVID-19 knows no borders, spares no country or continent, and strikes indiscriminately [3,4].

As COVID-19 sweeps the world, people are prone to panic, anxiety, and demonstrate lack of rationality, and rumors and misinformation about COVID-19 spread among the masses, referred to as an “infodemic”. During the SARS epidemic in 2003, “infodemic” was adopted by health analyst Rothkopf to describe the situation at that time: fact, fear, speculation, and rumors were mixed and were rapidly amplified and spread worldwide by modern information technology [5,6]. The impacts of the infodemic on the national economy, international politics, and even security are far more profound than that of the epidemic itself [7]. The outbreak of COVID-19 was followed by the situation of infodemic. As Sylvie Briand, Director of Global Infectious Disease Prevention of the WHO, pointed out, in order to actively respond to COVID-19, it is necessary to respond to the infodemic. She defined an information epidemic as the fact that too much information (both true and false) makes it difficult for people to identify the authenticity, find reliable information, and obtain effective guidance, which even harms people’s health [8].

In the context of the infodemic, media organizations play an important role. On the one hand, they should convey correct knowledge of epidemic prevention and guide the public to protect themselves from COVID-19 [9]; on the other hand, they should disseminate the true situation of the epidemic to the public. Therefore, the objectivity and impartiality of the news media are particularly important [10,11].

Benjamin Franklin’s practice of taking over the Pennsylvania Gazette has made outstanding contributions to the germination of the principle of objectivity in news media organizations. It mainly refers to “When people have different opinions on something, both sides have equal opportunities to explain their ideas” [12]. Meanwhile, journalists should not falsify the facts or fool the public with personal prejudice and interests. However, the concept of objectivity has stimulated much-complicated discussion and controversy in journalism [13]. Some scholars have pointed out that there was no attainability of objectivity [14]. Reporters and editors are conditioned by many factors such as gender, circumstance, and education [15]. Therefore, there is an increasing acceptance of subjectivity across different forms of journalism [16]. On the contrary, some scholars hold the perspective that the principle of objectivity still applies in journalism. Despite the above criticism, however, objectivity is still believed to be the almost only form of “good” journalism [17]. The norm of news objectivity essentially involves just two epistemic norms: factual accuracy and justified interpretation, which means objectively verified facts are just statements that can be believed as true based on evidence [18]. Impartiality in journalism also shows a lofty status in the Western press. Compared to objectivity, some journalists are even more concerned with impartiality. Respecting facts, eliminating bias, and paying attention to the social significance of news are important factors to ensure impartiality [19]. The above definition shows that impartiality requires journalists to attach importance to eliminating various prejudices and to accurately report the news based on facts. Therefore, during the epidemic, media organizations should also truthfully report the true situation of the epidemic in various countries based on the principle of impartiality.

In the field of media and communication, during the epidemic, scholars paid more attention to using the remarks made by social media Internet users as research materials and study from sentiment analysis and topic modeling. Most scholars have studied the emotional changes in people during the epidemic from the perspective of sentiment analysis. Wang et al. randomly selected 999,978 COVID-19-related Weibo posts and applied them in the unsupervised bidirectional encoder representations from transformers (BERT) model for sentiment analysis [20]. Some scholars were concerned more with topic modeling and use topic modeling methods such as BERT or LDA to study the topics that the masses are concerned about during the epidemic. Jelodar et al. extracted COVID-19–related discussions from social media and adopted a natural language process (NLP) method based on topic modeling to uncover various issues related to COVID19 from public opinions. Moreover, they investigated how to use the long and short-term memory (LSTM) recurrent neural network for sentiment classification of COVID-19 comments [21].

Based on the above content, it can be concluded that current research in media and communication during the COVID-19 pandemic mainly focuses on discussions of the masses and the posts of Internet users. There is less research focusing on the reports of news agencies during the pandemic. Not only that, but a few scholars have also tried to quantify the impartiality of media into indicators and compare the impartialities of different media on the anti-epidemic performance of different countries.

Therefore, our research deeply analyzes the impartiality of different media and is guided by the following specific questions:

Q1: Is the coverage of the COVID-19-related news about different country objective, according to the anti-epidemic performance of different countries?

Q2: Is the impartiality of the media related to its reliability?

Q3: Is the impartiality of the media related to its political bias?

Q4: Are there significant differences in media beliefs in different countries when they report the domestic situation of different countries during COVID-19?

The following conclusions were drawn after crawling news, cleaning the data, analyzing the sentiment of the text with the SentiWordNet3.0 and BERT models, and, finally, performing a series of steps such as data visualization.

- Judging from the media’s news on the anti-epidemic performance of various countries, we found the sentiment of the media’s COVID-19-related news about certain countries did not always match their anti-epidemic performance;
- The impartiality of the media is weakly related to its reliability;
- The impartiality of the media is not related to its bias;
- There are obvious differences in the willingness and positiveness of international media to report on the domestic performance of different countries during the COVID-19 pandemic.

## 2. Materials and Methods

**Figure 1:**
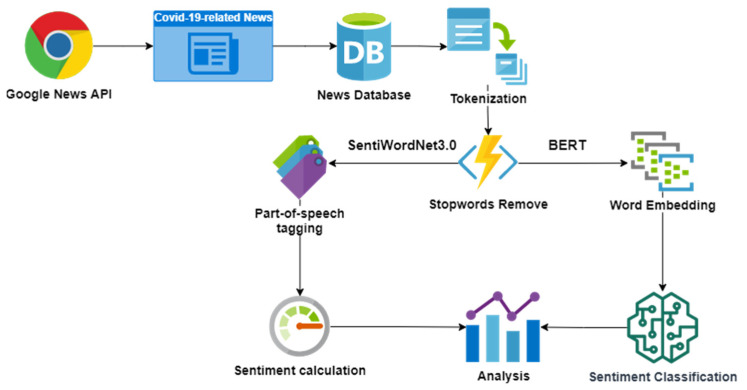
The Flowchart of Data Collection and Sentiment Analysis.

As shown in Figure 1, we collected COVID-19-related news articles from several news agencies using the Google news API. We presented a database for sentiment analysis after eliminating duplicate and missing data. First, we tokenized the text of news articles and removed stop words. Then, we adopted two types of sentiment analysis techniques: Sentiment analysis based on SentiWordNet3.0 and BERT. In the SentiWordNet3.0-based sentiment analysis, we tagged each part of speech and evaluated its sentiment value. In the BERT-based sentiment analysis, words were embedded using the Global Vectors for Word Representation (GloVe) and the BERT model of sentiment classification was trained. Finally, we obtained the outcome of sentiment analysis for subsequent investigation.

### 2.1. Materials

To solve the problems raised in the introduction, the COVID-19 dataset and COVID-19-related news dataset are set up here.

#### 2.1.1. The COVID-19 Dataset

The numbers of infected cases and deaths caused by COVID-19 in ten countries (including the United States of America, China, Spain, Italy, Korea, Russia, India, the United Kingdom, Singapore, and Japan) from 1 January 2020 to 30 September 2020 were collected from the dataset published on the official website of the WHO.

#### 2.1.2. The COVID-19-Related News Dataset

With the help of Google News API, we searched news agencies’ websites with “COVID-19 + Country” as the keyword to obtain the URLs of COVID-19-related news in English. To obtain a response from URLs, we crawled COVID-19-related news headlines, release time, and text. Before the data are finally used in sentiment analysis, it should be cleaned. So, we removed useless data that were empty or duplicated. Finally, we obtained 38,367 pieces of COVID-19-related news from 18 news agencies, including ABC (ABC News), CD (Common Dreams), CNN, Fair, Fortune, Fox, OANN (One America News Network), RT (Russia Today), SN (Sputnik News), VOA (Voice of America), WSTE (Washington Examiner), BBC (British Broadcasting Corporation), NYT (New York Times), CGTN (China Global Television Network), TAS (The Asahi Shimbun), HT (Hindustan Times), KJD (Korea Joongang Daily), and TST(The Straits Times).

### 2.2. Methods

#### 2.2.1. Sentiment Analysis with SentiWordNet3.0

In our research, SentiWordNet3.0 is adopted for sentiment analysis. SentiWordNet 3.0 is an improved version of SentiWordNet 1.0, a lexical resource publicly available for research, currently licensed to more than 300 research groups and used in a variety of research projects worldwide [22]. SentiWordNet3.0 is devised for supporting sentiment classification and opinion mining. During this process, the semi-supervised learning and the random-walk are adapted to process the input text. To better perform the subsequent sentiment analysis, SentiWordNet3.0 normalizes Pos(*s*) and Neg(*s*), which are given in Equation (1):

(1) $$\mathrm{Pos}(s)\prime =\frac{\mathrm{Pos}(s)-\min(\mathrm{Pos}(s))}{\max(\mathrm{Pos}(s))-\min(\mathrm{Pos}(s))}$$

where, Pos(s)′ and Pos(s) refer to the positive value of the word after the normalization semi-supervised learning, respectively.

The text is imported and then segmented. The sentence is made up of punctuation marks, spaces, and words. Thus, the sentence is divided into several words according to the spaces and punctuation marks. Later, the text is processed with the help of natural language toolkits (NLTKs) stop word corpus consisting of the words that appear frequently but have little actual meaning, such as personal pronouns, modal particles, and prepositions. Eliminating stop words can effectively improve the efficiency of text sentiment analysis. After the above steps are completed, the input text becomes an array of words. Then, the Part-Of-Speech tagging (POS tagging) is performed after segmentation, which is one of the important steps of text data preprocessing. The high-level features can be obtained from original text through POS tagging in NLP or text mining applications. After the POS tagging, it will be convenient for input to the parser to perform the sentiment analysis [23].

Next, the most important step is to calculate the sentiment score of the word. Because a word may be composed of multiple parts of speech, and each part may have multiple meanings, the sentiment value of the word can be obtained by weighting all the scores of the word. In SentiWordNet, the higher the order of the meaning within a part of speech of a word, the more the meanings can represent. Thus, 1 and 2 are given to the first and the second meaning, respectively. Among them, the sentiment score of each word is the positive score minus the negative score, as shown in Equation (2). Finally, the sentiment score of the entire text is calculated based on the number of words and word score.

(2) $$\mathrm{Score}(s)=\frac{\sum_{1}^{n}(\mathrm{Pos}(s)-Neg(s)){\frac{1}{2}}^{n-1}}{n}$$

where, Score(s) is the emotional value of the word, Pos(s) is the positive value of the word, Neg(s) refers to the negative value of the word, and n is the number of the word’s meanings.

#### 2.2.2. Sentiment Analysis with BERT

To study the coverage of international news agencies of other countries, articles issued by China Global Television (CGTN) and other international news agencies are crawled and then performed with the sentiment analysis by using the BERT model. BERT is a language representation model, which is trained through super-large data, a huge model, and huge computational overhead. It has achieved the state-of-the-art in 11 natural language processing tasks [24]. BERT aims to pre-train the deep bidirectional representation by joint adjustment of the left and right contexts in all layers. Therefore, only an additional output layer is required to fine-tune the pre-trained BERT representation to create the state-of-the-art models for a wide range of wider tasks, such as language inference, without so many task-specific models. Therefore, the finetuned BERT model is adopted in our research for sentiment analysis of articles.

A total of 2000 articles are randomly extracted to build the training dataset and the test dataset. The extracted text is classified into two categories by manual labeling: 1 for positive texts and 0 for negative ones. Next, the samples are randomly selected at a ratio of 3:1 to form the training dataset and the test dataset. After that, a fine-tuned BERT model is built: the Bert model and the linear layer participated in training together, making the Bert model more suitable for the classification of our research.

Firstly, the original training dataset is processed and divided into a training dataset in 64 batch-size, so that the model can perform batch gradient descent while reducing the operating pressure of the system. Then, the BERT corpus is incorporated for feature extraction on the training text, and then a linear layer is connected to the (CLS) output position of the BERT model to predict the classification of sentences. Finally, termination of model training has to be considered when the loss function reaches the optimum, to avoid overfitting.

After the model training is completed, the finetuned BERT model is employed to classify the test dataset, and the accuracy of the fine-tuned BERT model is calculated. In addition, an LR model and a support vector machine (SVM) model are constructed for sentiment classification, and the accuracy is calculated accordingly. Table 1 shows that the accuracy of the fine-tuned BERT model is higher than that of the other two models.

## 3. Results

### 3.1. The Relationship between Sentiment of the COVID-19-Related News and the Data

**Figure 2:**
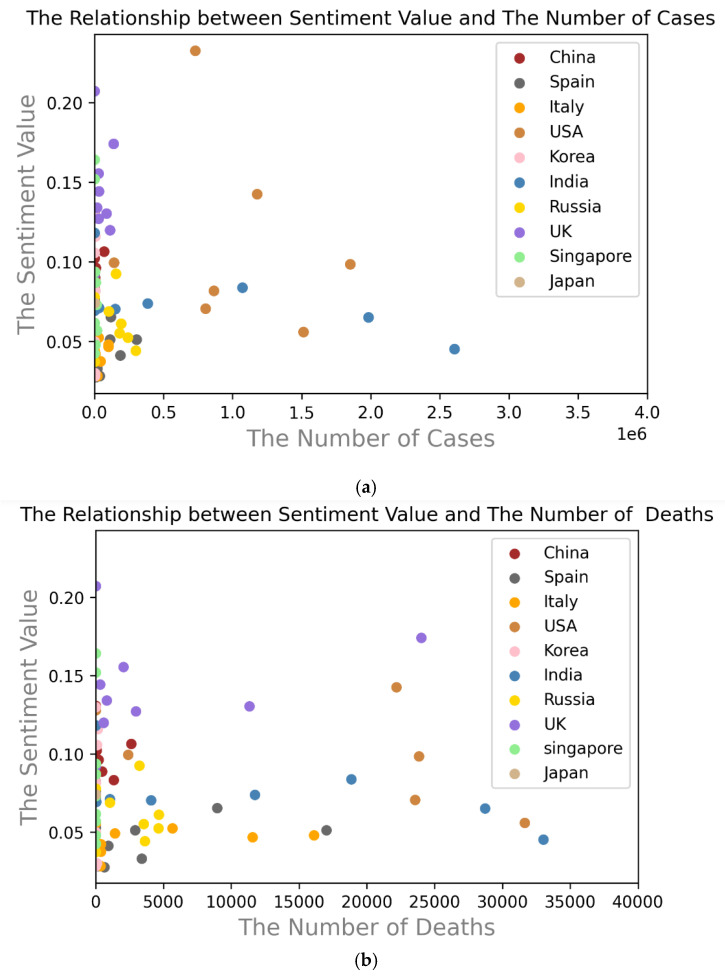
(**a**) The relationship between sentiment and the number of cases in different countries; (**b**) The relationship between sentiment and the number of deaths in different countries.

Texts of COVID-19-related news published by various news agencies in different countries are searched by Google News and put into the sentiment analysis system based on SentiWordNet 3.0 to obtain their sentiment values. To rigorously prove whether the news reports are in line with the true situation, two scatter plots are proposed in our research: one is a scatter plot of the number of confirmed cases and the sentiment values, and the other is a scatter plot of the number of deaths and sentiment values, as shown in Figure 2. As illustrated in Figure 2a,b, there is no obvious correlation between the distribution of points in the two scatter plots.

After that, Pearson correlation analysis, as shown in Equation (3), is adopted to analyze the association of the three variables above, including the number of confirmed diagnoses, the number of deaths, and the sentiment value, as shown in Table 2.

(3) $$r=\frac{\sum \left(x_{i}-\bar{x}\right)\left(y_{i}-\bar{y}\right)}{\sqrt{\sum {\left(x_{i}-\bar{x}\right)}^{2}\sum {\left(y_{i}-\bar{y}\right)}^{2}}}$$

where, r is the Pearson correlation coefficient, $x_{i}$ is sentiment values, $\bar{x}$ is the averaged sentiment values, $y_{i}$ is the number of cases/deaths, and $\bar{y}$ is the average of the number of cases/deaths.

As shown in Table 2, the Pearson coefficient between the number of cases and the sentiment value is 0.02, and that between the number of deaths and the sentiment value is 0.19. When the Pearson coefficient is 0.0~0.2, the two show no correlation or a very weak correlation, indicating no correlation between the reported sentiment value and the number of confirmed cases. The above content shows that the news failed to reflect the true epidemic in various countries. Based on the above research findings, our research further studies the factors affecting the impartiality of news agencies.

### 3.2. The Factors Affecting the Impartiality of News Agencies

To study the factors affecting the impartiality of news agencies, it is important to distinguish and quantify the attributes of media. Tim et al. quantified the attributes of media and confirmed it could be divided into media reliability and bias [25]. In our research, 13 news agencies are enrolled, including ABC (ABC News), CD (Common Dreams), CNN (Cable News Network), Fair, Fortune, Fox, OANN (One America News Network), BBC (British Broadcasting Corporation), NYT (New York Times), RT (Russia Today), SN (Sputnik News), VOA (Voice of America), and WSTE (Washington Examiner). The reliability and bias of news agencies are shown in Table 3.

To study the impacts of different attributes on the impartiality of media, news agencies with different attributes are selected as the research objects in our research.

In this part, the proportion of positive articles that report on a country’s epidemic situation is adopted to measure the sentiment of a media organization towards the country. This is because, compared to using the average sentiment value, the proportion of articles with positive sentiment can better reflect the sentiment tendencies reported by the news agency during the epidemic. To achieve the above goals, the sentiment of the text should be classified firstly according to the sentiment value of the article, which can be positive, neutral, or negative, as shown in Equation (4). When the s value is greater than 0.05, it should be classified in the positive category. Then, the above criteria are applied to classify all news from the media.

(4) $$TS(CS)=\left\{\begin{array}{ll} \;\mathrm{positive}\; & CS\geq 0.05\\ \;\mathrm{negative}\; & CS\leq -0.05\\ \;\mathrm{neutral}\; & \;\mathrm{otherwise}\; \end{array}\right.$$

where, TS is the category of the article’s sentiment, and CS is the article’s sentiment value.

After the article sentiment classification, the proportion of positive texts in all articles of the news agencies should be calculated. According to Equation (5), the sentimental proportion of articles on epidemics in different countries can be obtained.

(5) $$P_{\mathrm{pos}\;}=\frac{N_{\mathrm{pos}\;}}{N_{\mathrm{pos}\;}+N_{neu}+N_{neg}}$$

where, $P_{\mathrm{pos}\;}$ is the proportion of positive texts in all articles of the news agencies, $N_{\mathrm{pos}\;}$ is the number of positive articles, $N_{neu}$ is the number of neutral articles, and $N_{neg}$ is the number of negative articles.

**Figure 3:**
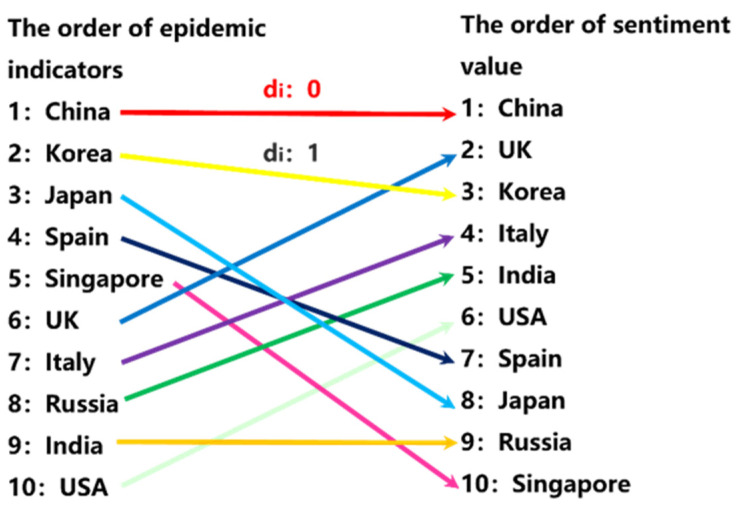
The schematic diagram of ranking difference.

As demonstrated previously, when a news agency follows the principle of impartiality, the proportion of positive articles for different countries should be more in line with the real situation of the country during COVID-19. In Figure 3, the left side shows the ranking of epidemic indicators, and the right side displays the ranking of the proportion of positive articles. In a completely fair media, the ranking of the proportion of positive articles should be completely the same as the ranking of epidemic indicators. The ranking difference can be referred to by using di. The larger the ranking difference, the greater the difference between the media’s coverage of the situation of the country dealing with the epidemic and the reality.

To study the impartiality of the news agencies, our research attempts to use the Spearman coefficient to quantify the impartiality of the news agencies, as shown in Equation (6). The Spearman coefficient can fully reflect the ranking difference between the two variables, so it can measure the difference between a media’s report on each country and the real situation. Its value is −1~1. The closer to −1, the greater the difference between the media’s report and the real situation.

(6) $$\rho =1-\frac{6\sum d_{i}^{2}}{n\left(n^{2}-1\right)}$$

where, ρ is the Spearman coefficient of the ranking of the proportion of positive articles and the ranking of COVID-19’s indicators, $d_{i}^{}$ is the ranking difference, and n is the number of the reported countries.

**Figure 4:**
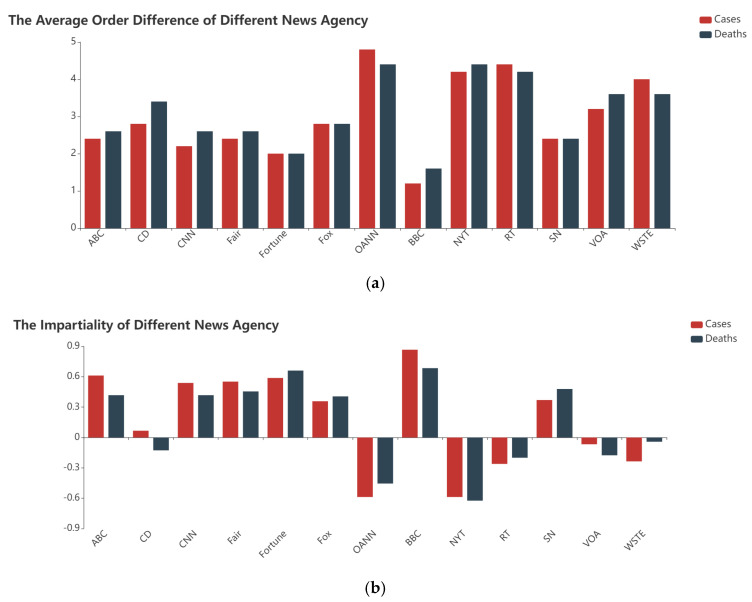
(**a**) The average ranking difference of different news agencies; (**b**) The impartiality of different news agencies.

Based on the above steps, the average ranking difference and fairness of the 14 media are calculated based on the number of confirmed cases and the number of deaths, and the obtained results are illustrated in Figure 4. Figure 4a reveals that the average ranking difference of these news agencies is 2~5 and is similar to each other. Therefore, it is difficult to determine which media reports are more in line with the real situation with the average ranking difference. Figure 4b discloses that the differences in the impartiality of news agencies are obvious. The impartialities of the BBC are significantly higher than those of other media, and those of OANN are significantly lower than those of other media.

To analyze the factors affecting the news agencies’ impartiality, a correlation analysis is performed on the reliability and the bias of news agencies with the impartiality calculated based on the number of confirmed cases and the number of deaths, as shown in Table 4.

The correlation between the affecting factors and the news agencies’ impartiality can be expressed as Equation (7) below.

(7) $$H(S)=\left\{\begin{array}{ll} \;\mathrm{Very}\;\mathrm{strong}\;\mathrm{correlation}\; & S\geq 0.8\\ \;\mathrm{Strong}\;\mathrm{correlation}\; & S\geq 0.6\\ \;\mathrm{Medium}\;\mathrm{degree}\;\mathrm{correlation}\; & S\geq 0.4\\ \;\mathrm{Weak}\;\mathrm{correlation}\; & \;\mathrm{otherwise}\; \end{array}\right.$$

where, H(S) is the correlation of the affecting factors and the news agencies’ impartiality, and S is the Pearson correlation coefficient.

Combined with the values in Table 4, the reliability of news agencies has a medium degree correlation with the impartiality calculated based on the number of confirmed cases, and a weak correlation with the impartiality calculated based on the number of deaths. However, the bias of news agencies shows no obvious relationship with the impartiality calculated based on the number of confirmed cases or the number of deaths.

Therefore, in COVID-19-related news, the reliability of the news agencies affects the impartiality of the media; that is, the higher the reliability of the media, the more truthful the articles are. The bias of the media shows no obvious impact on impartiality; that is, there is no significant difference in media coverage on COVID-19, although they have different political leanings. In the infodemic caused by COVID-19, the news agencies with high reliability are more likely to disseminate the true epidemic situation and shoulder the responsibility of eliminating misinformation.

### 3.3. The Differences in the Willingness and Positiveness of International Media Coverage in Different Countries

**Figure 5:**
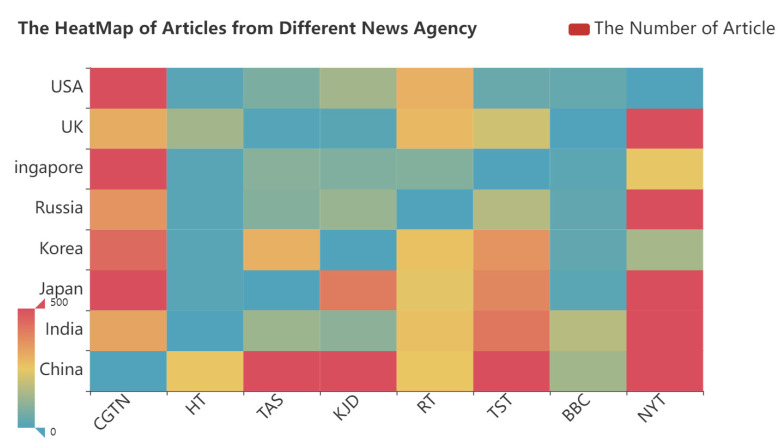
The number of COVID-19-related articles from news agencies reporting on different countries.

**Figure 6:**
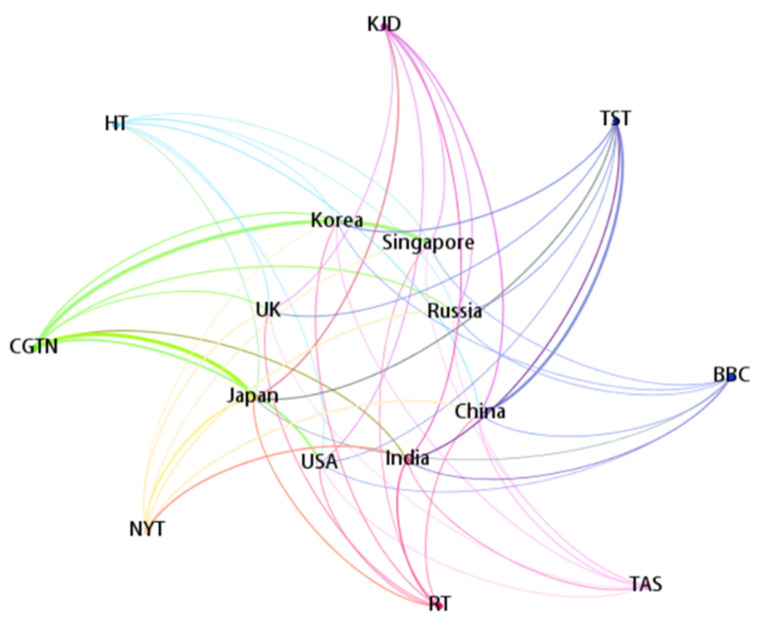
The number of COVID-19-related articles be positive.

The fine-tuned BERT model is employed to classify all the articles. After the classification results are obtained, our research processes the total number of articles from the selected media and the articles judged to be positive about the COVID-19 situation in different countries. Based on the data, a heatmap of the total number of COVID-19-related articles about different countries and a network of positive reports are proposed, respectively, as shown in Figure 5 and Figure 6.

According to Figure 5, different news agencies occupy different total numbers of reports. Among them, the number of countries reported by CGTN and NYT is significantly higher than that reported by other news agencies, while other countries such as RT and the BBC, report significantly lower volumes. In addition, obvious differences are found in the number of media reports in different countries. The above data show that during the COVID-19 pandemic, there were obvious differences in the amount of coverage of international news agencies, which impacted the public’s impression of the severity of COVID-19 in different countries.

According to Figure 6, NYT, TST, and CGTN have significantly higher numbers of positive COVID-19-related reports on other countries than other news agencies. The media are gatekeepers, so the positive coverage of the pandemic by these media will effectively improve the performance of these countries in fighting COVID-19 in the eyes of the masses.

However, the above two figures can only partly reflect the bias of these media to target different countries. To further study the tendency of the media to report on various countries, our research tries to learn from ZAHID [26] and measures the absolute coverage score (ACS) and absolute positive score (APS) of each media. Based on the above data, the ACS and APS of each media for each country’s COVID-19-related articles can be obtained, and then a scatter chart can be drawn, as shown in Figure 6.

**Figure 7:**
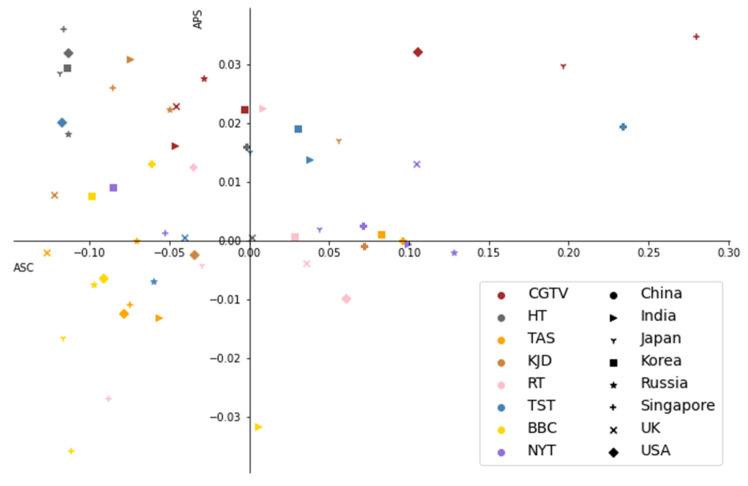
The ACS and APS of international news agencies.

As demonstrated in Figure 7, there are huge differences in the coverage scores and positive scores of COVID-19-related reports by different media in various countries. The coverage scores and positive scores of the BBC are both low, so articles on various countries account for a relatively small proportion, and the BBC tends to carry out negative reports, especially for Singapore and Japan. The coverage score of NYT is low, but the proportion of positive reports for each country is relatively high, indicating that NYT is the same as the BBC in terms of the number of reports on the pandemic situation in each country, but it is more inclined to make positive comments on some countries such as the United Kingdom and Singapore.

Based on the above description, it can be concluded that there are obvious differences in the willingness and positiveness of international media to report on the performance of different countries during the COVID-19 pandemic.

## 4. Discussion

Using sentiment analysis of COVID-19-related news about various countries, we examined the impartiality of news agencies and their factors in the present study. According to the situation of COVID-19 in different countries, the results indicate that the news coverage of COVID-19 in different countries is not objective. Previous studies have examined the media coverage of COVID-19 in different countries. In an ongoing study of the soundtracks from Western news coverage of the pandemic at the first major sites of infection and quarantine, such as Wuhan (China), Tehran (Iran), and Milan (Italy), researchers have found that the distribution of sound, silence, and music differs regionally and nationally [27]. Meanwhile, mainstream news organizations’ social media posts of different countries maintain a strong elite orientation [28].

We further investigate the factors of media coverage of various nations, or the factors of media impartiality. We tentatively conclude that the reliability of the media has a weak correlation with its impartiality and that there is insufficient evidence to establish a connection between media’s impartiality and political bias. There is a significant amount of discussion among scholars about the principle of impartiality and objectivity in communication studies [15,16]. The qualitative methods are widely applied to study the impartiality of media and its factors. Our research may provide a possible quantitative method for research on the impartiality of media.

It seems that there are obvious differences in the willingness and positiveness of international media in different countries to report on different countries’ performance during the COVID-19 pandemic. In previous studies, the sentiment or emotion of COVID-19-related news of media in different countries turned out to have clear differences. Emotions such as sadness and anger were detected in Korean broadcasters, whereas those emotions were not detected in the US broadcasters [29].

One of the limitations in this study is that the accuracy of the sentiment analysis models needs to be improved, for we only built a training library of 2000 texts. Meanwhile, we concentrate on only two possible factors of the media’s impartiality (the reliability and political bias of media). The economic composition of the media and the personal writing style of journalists are also important factors.

With the development of the Internet, the exchange of information around the world has become more frequent, especially on some important global issues, such as the COVID-19 pandemic. Our research provides a good starting point for discussion and further research about the performance of media on an important topic.

## 5. Conclusions

In the present study, we found that the coverage of COVID-19-related news about different countries is not objective. Additionally, we tentatively infer that the reliability of the media has a weak correlation with its impartiality. It seems that there are obvious differences in the willingness and positiveness of international media in different countries to report on different countries’ performance during COVID-19.

In communication science, the impartiality and objectivity of media and its factors have been discussed by many scholars, mainly in the qualitative method. Our research may provide a possible quantitative method for research on the impartiality or the objectivity of media, especially based on the sentiment analysis model. Meanwhile, the potential effects of other factors should be considered carefully, for example, the personal characteristics of journalists.

Looking forward, further attempts could prove quite beneficial to incorporate the method in media literacy programs. Due to some important global issues such as the COVID-19 pandemic, it is necessary to find the media with high impartiality as a reliable source of information. With the help of the method based on sentiment analysis, people can improve their media literacy and accept more objective information than before.

## Figures and Tables

**Table 1:** The accuracy of models.

Model	Accuracy
LR	0.654
SVM	0.674
BERT	0.710

**Table 2:** The Pearson correlation between the sentiment values and the number of cases/deaths.

	The Number of Cases	The Number of Deaths	The Sentiment Values
The Number of Cases	1.00	0.76	0.02
The Number of Deaths	0.76	1.00	0.19
The Sentiment Values	0.02	0.19	1.00

**Table 3:** The reliability and political bias of news agencies.

News Agency	Reliability	Bias
ABC	48.22	−4.79
CD	41.29	−17.80
CNN	44.20	−10.13
Fair	34.76	19.34
Fortune	44.85	0.17
Fox	31.62	17.78
OANN	21.63	21.95
BBC	46.18	−2.72
NYT	42.96	−7.67
RT	30.99	14.32
SN	37.18	9.79
TMZ	39.52	−10.41
VOA	47.43	−5.44
WSTE	25.20	17.83

**Table 4:** The Pearson correlation between the sentiment values and the number of cases/deaths.

	Reliability	Bias	Impartiality Based on Cases	Impartiality Based on Deaths
Reliability	1.00	−0.84	0.54	0.36
Bias	−0.84	1.00	−0.21	−0.01
Impartiality based on cases	0.54	−0.21	1.00	0.96
Impartiality based on deaths	0.36	−0.01	0.96	1.00

## Data Availability

Data presented is original and not inappropriately selected, manipulated, enhanced, or fabricated.
